# Design and Optimization of the Single-Stage Continuous Mixed Suspension–Mixed Product Removal Crystallization of 2-Chloro-*N*-(4-methylphenyl)propenamide

**DOI:** 10.1021/acsomega.1c07228

**Published:** 2022-04-13

**Authors:** Gladys Kate Pascual, Philip Donnellan, Brian Glennon, Barbara Wood, Roderick C. Jones

**Affiliations:** † Synthesis and Solid State Pharmaceutical Centre (SSPC), School of Chemical and Bioprocess Engineering, University College Dublin, Belfield, Dublin 4, Ireland; ‡ APC Ltd, Cherrywood Business Park, Loughlinstown, Dublin D18 DH50, Ireland

## Abstract

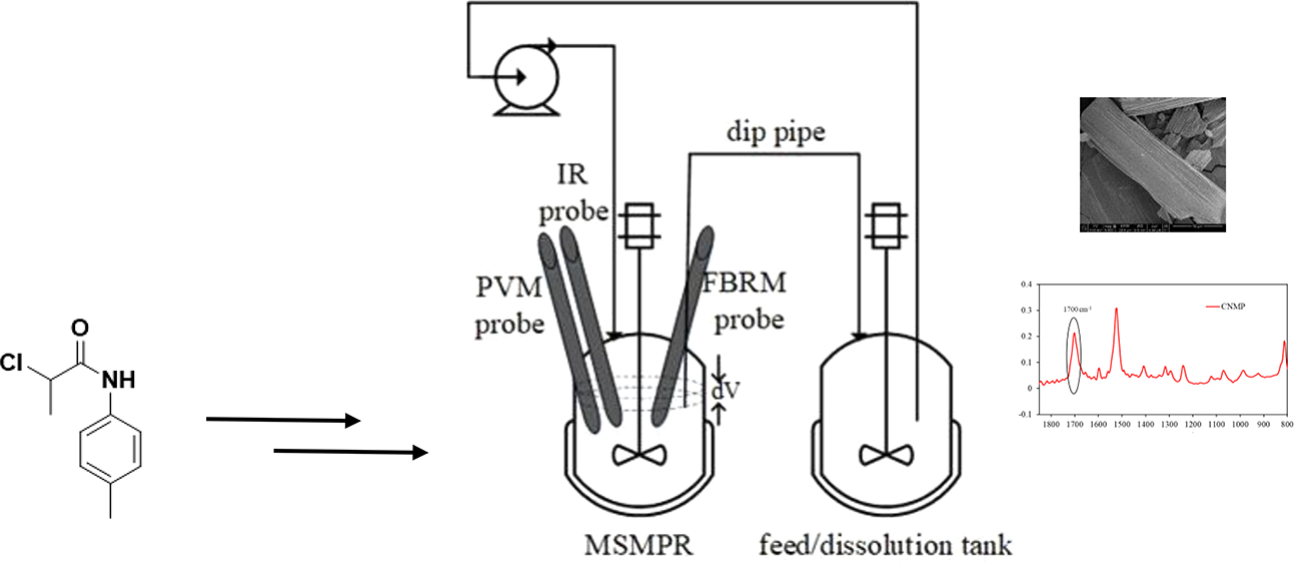

A continuously operated single-stage mixed suspension–mixed product removal (MSMPR) crystallizer was developed for the continuous cooling crystallization of 2-chloro-*N*-(4-methylphenyl)propanamide (CNMP) in toluene from 25 to 0 °C. The conversion of the previous batch to a continuous process was key to developing a methodology linking the synthesis and purification unit operations of CNMP and gave further insight in the development of continuous process trains for active pharmaceutical ingredient materials. By monitoring how parameters such as cooling and agitation rates influence particle size and the yield, two batch start-up strategies were compared. The second part of the study focused on developing and optimizing the continuous cooling crystallization of CNMP in the MSMPR crystallizer in relation to the yield by determining the effects of varying the residence time and the agitation rates. During the MSMPR operation, the plot of the focused beam reflectance measurement total counts versus time oscillates and reaches an unusual state of control. Despite the oscillations, the dissolved concentration was constant. The yield and production rate from the system were constant after two residence times, as supported by FTIR data. The overall productivity was higher at shorter residence times (τ), and a productivity of 69.51 g/h for τ = 20 min was achieved for the isolation of CNMP.

## 1 Introduction

Continuous processing is becoming increasingly common in the pharmaceutical industry and offers numerous benefits for safety, productivity, and isolation in the synthesis of drug substances and active pharmaceutical ingredients (APIs).^1−5^ The implementation of continuous processing methods is taking place in both academia and industry, giving safe access to “forbidden” (traditionally toxic or exothermic) chemistry, multiphasic synthesis, and real-time reaction interrogation and intervention through the application of online procession analytical tools.^6−11^ In this way, reaction pathways deemed too operationally difficult or dangerous in conventional batch equipment can be developed. As the API product is constantly removed from potentially compromising environments (often improving the purity of the isolated molecule), benefits can be identified not only in synthesis steps but also in the optimization of downstream unit operations, leading to increases in productivity and efficiency through the telescoping of these processes.^12,13^ A major focus of this research is the development of design and optimization strategies that deliver robust, tunable, and scalable API manufacturing processes, delivering specific API characteristics.^14−18^

**Figure 1:**
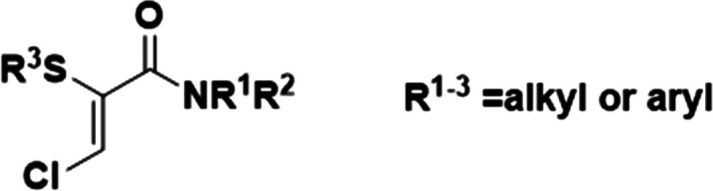
Generalized structure of α-thio-β-chloroacrylamides.

**Scheme 1:**
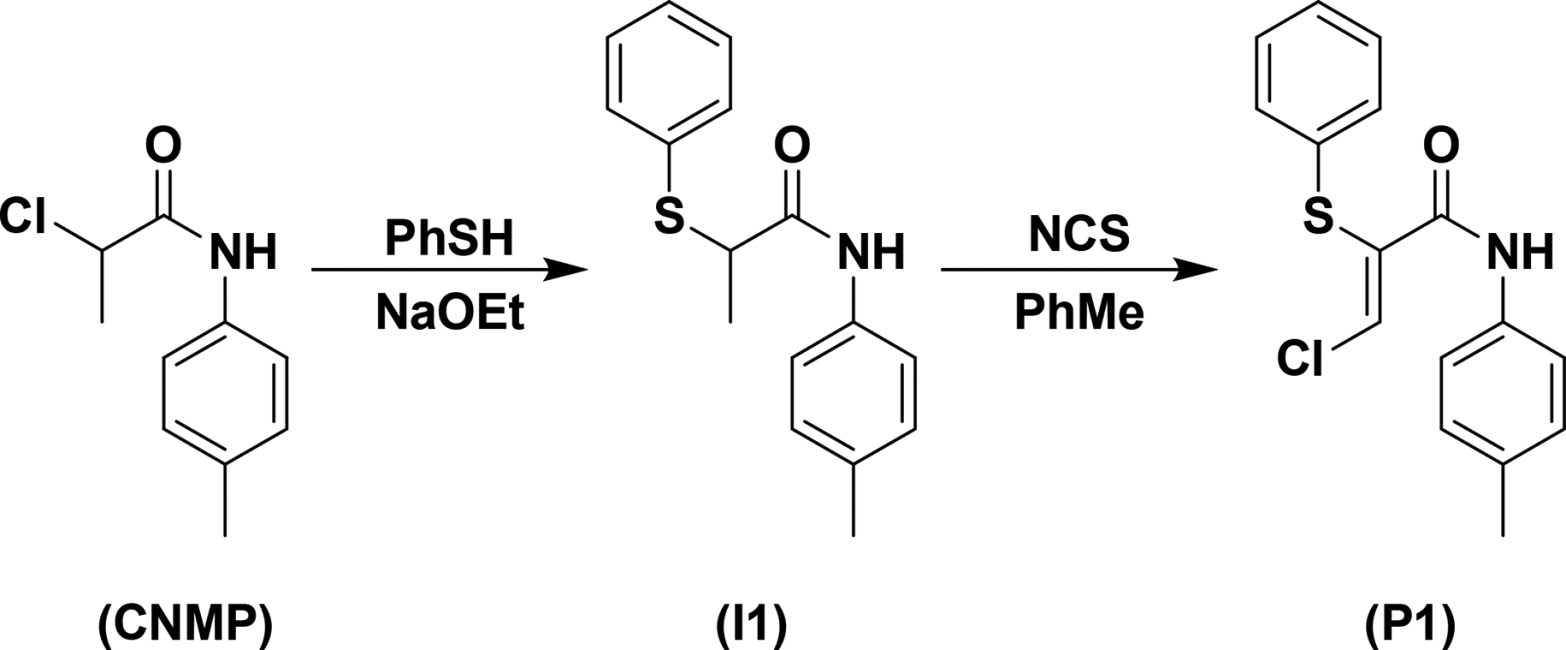
Synthesis of (*Z*)-3-Chloro-2-(phenylthiol)-*N*-(*p*-tolyl)acrylamide (P1)

The synthesis and crystallization of 2-chloro-*N*-(*p*-tolyl)propanamide (CNMP),^19,20^ a key starting material in the synthesis of α-thio-β-chloroacrylamides (Figure 1) and a synthetically important API intermediate, has been shown to undergo a number of fundamental synthetic transformations, such as 1,3-dipolar cycloadditions,^21^ [3 + 2]-dipolar cycloadditions,^22^ Diels–Alder cycloadditions,^23^ and nucleophilic^24^ and sulfide group substitutions.^25^ β-Chloroacrylamides can have overall complex cascade synthetic pathways, and the desired product purity profiles are highly dependent on the starting material’s purity. Consequently, CNMP, a precursor in the multistep synthesis of (*Z*)-3-chloro-2-(phenylthio)-*N*-(*p*-tolyl)acrylamide (**P1**, an API of interest to this group, Scheme 1), requires extensive purification via crystallization before use in order to obtain clean product profiles.

A goal of the research was to design and develop a continuous crystallization process for CNMP for the large-scale synthetic purification and isolation of a precursor in the multistep synthesis of **P1** (an API of interest to this group, Scheme 1).

In the pharmaceutical industry, two commonly used continuous crystallizers are the mixed suspension–mixed product removal (MSMPR) crystallizer and the plug flow crystallizer (PFC).^26^ Two advantages of continuous processing for crystallization are the smaller-scale process equipment required and its smaller footprint (when compared to batch processes), which can translate into a decrease in the initial capital expenditure.^27^ Ferguson et al. demonstrated the process intensification capabilities of continuous crystallization when compared to batch crystallization and showed that the output of a 10 000 L batch system could be matched by the continuous operation of a 9 L MSMPR crystallizer or a 33 mL lab-scale PFC.^28^ The choice between a MSMPR crystallizer and a PFC is generally driven by the kinetics of the process, where MSMPR is generally favorable for compounds with slow kinetics, long conversions, and long residence times, while a PFC is preferred for compounds with fast kinetics, high conversions, and short residence times.^13^ Another influence on the choice between MSMPR and a PFC is that existing batch infrastructure can be used for MSMPR operation, giving a lower capital expenditure when compared to the PFC.^13^

Based on the solubility studies performed for CNMP in toluene, cooling crystallization can provide a reasonable theoretical yield of 70% when a saturated solution at 25 °C is cooled to 0 °C and fully de-supersaturated.^20^ Performing cooling crystallization from toluene would eliminate any requirement for a solvent exchange step operation between the continuous synthesis reaction and the crystallization process, enabling a simpler integration of the two steps. MSMPR crystallization was identified as the most suitable option for this process not only to avoid coating issues that could arise in a plug flow or continuous oscillatory baffled crystallizer (COBC) cooling crystallization process but also due to the anticipated requirements for the kinetics of the system to attain a high yield. The MSMPR is amenable to hold and surge points, and slight changes in the feed supply rate and the residence times would be expected to have a low impact on the yield and the particle size.^13^

To design a suitable MSMPR crystallization step for CNMP, several process parameters were investigated. While some constraints to integrate this step with the previous continuous synthesis step were present, i.e., the feed flow rate and the solvent selection of toluene, challenges around purity were not a consideration as no major impurities were identified after synthesis. Although CNMP is not a “model” compound, there were no challenges around the crystal form for crystallization. The main goal when developing the MSMPR step was optimizing the yield while simultaneously matching the throughput requirements of the previous continuous synthesis step. As an intermediate, the particle size distribution (PSD) requirements of CNMP were not specific except that the crystallized material be used for downstream isolation or forward processing as needed. An increase in residence time in the MSMPR should decrease the steady-state dissolved concentration (as the system is would allow more time for crystallization), leading to an increase in the yield.^16^ However, this can result in a decreased steady-state supersaturation impacting the kinetics of the system or indeed the kinetics of the system defining the supersaturation for different residence times. An increase in the agitation rate introduces higher fluid velocities and more violent crystal–impeller collisions, leading to a higher rate of secondary nucleation and a higher final crystallization yield.^29^ Therefore, different combinations of agitation rates and residence times were investigated to optimize the final yield from the system. The start-up procedure and steady-state attainment are important challenges in MSMPR continuous crystallization, as time and product can be wasted during this start-up period.^30,31^ Understanding the influence of the PSD from the batch start-up on the time it takes for the MSMPR crystallizer to attain a steady state is a key factor in optimizing the crystallization process. Consequently, two batch start-up strategies were selected for comparison to determine whether the PSD in the batch start-up would strongly influence the time taken for the MSMPR crystallizer to reach a state of control.

In the work outlined here, an efficient MSMPR step is designed that can continuously produce a consistent yield of CNMP for a subsequent redissolve or solvent swap step to feed the next reaction step in the synthesis of the API (*Z*)-3-chloro-2-(phenylthio)-*N*-(*p*-tolyl)acrylamide.

## 2 Experimental Section

### 2.1 Materials and Methods

#### 2.1.1 General Procedures

All solvents and reagents were commercially obtained from Sigma-Aldrich and used as received without further purification. For the synthesis of CNMP and spectral data of the obtained product,^19,20^ please see the SI.

### 2.2 Experimental Setup

**Figure 2:**
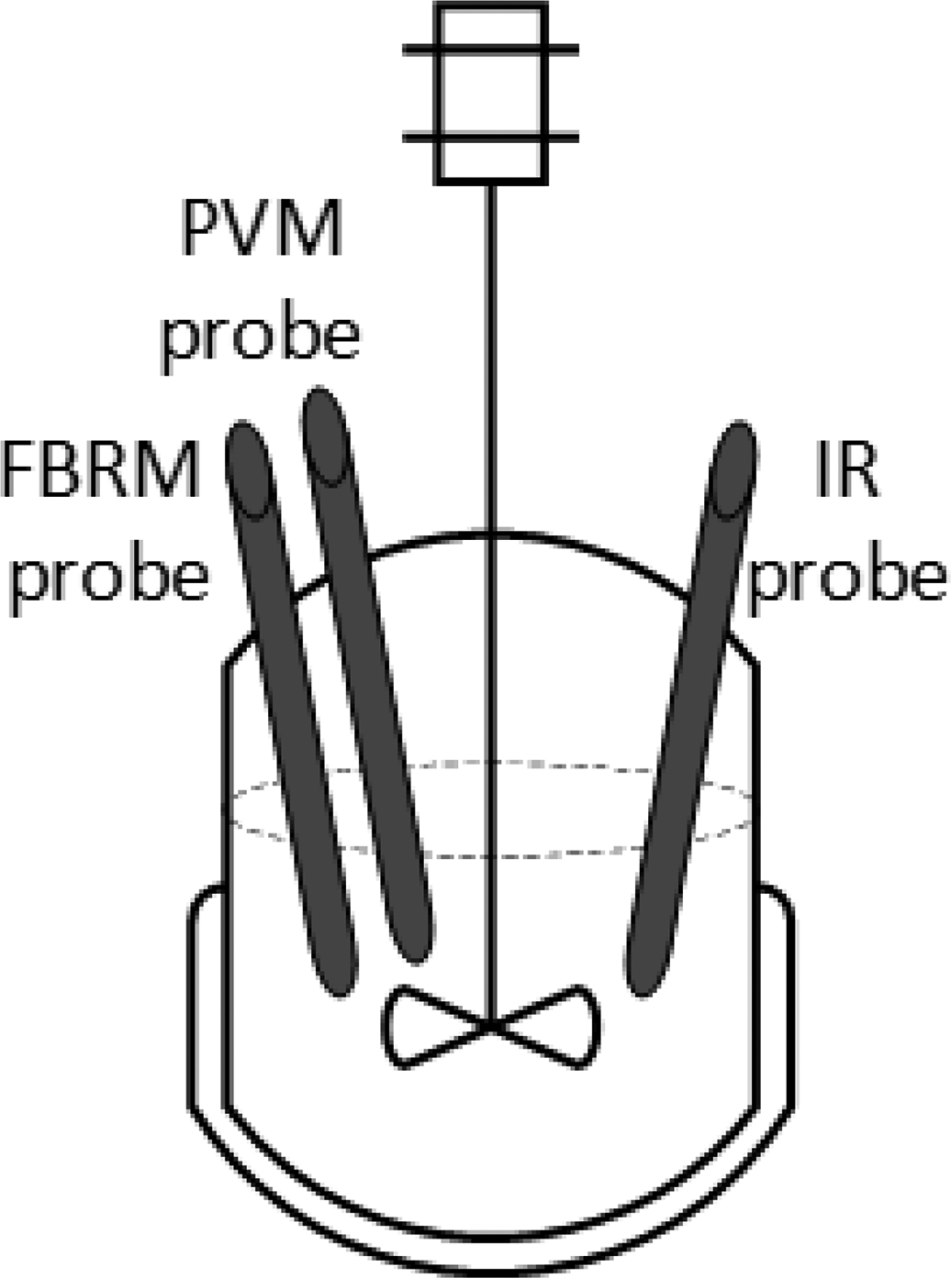
Schematic diagram of the experimental setup for batch studies.

Batch experiments were conducted using a Mettler Toledo OptiMax workstation equipped with a 1 L glass reactor and an agitator with steel pitch blade impeller (38 mm diameter with 45° inclined blades). The temperature of the crystallizer was controlled and monitored using iControl 5.5 (Mettler Toledo Software). Focused beam reflectance measurement (FBRM; ParticleTrack G400 series, Mettler Toledo), particle vision and measurement (PVM; V819 series, Mettler Toledo), and attenuated total reflectance fourier transform infrared spectroscopy (ATR-FTIR React IR 15, Mettler Toledo) were used to monitor the system during experiments (Figure 2).

**Figure 3:**
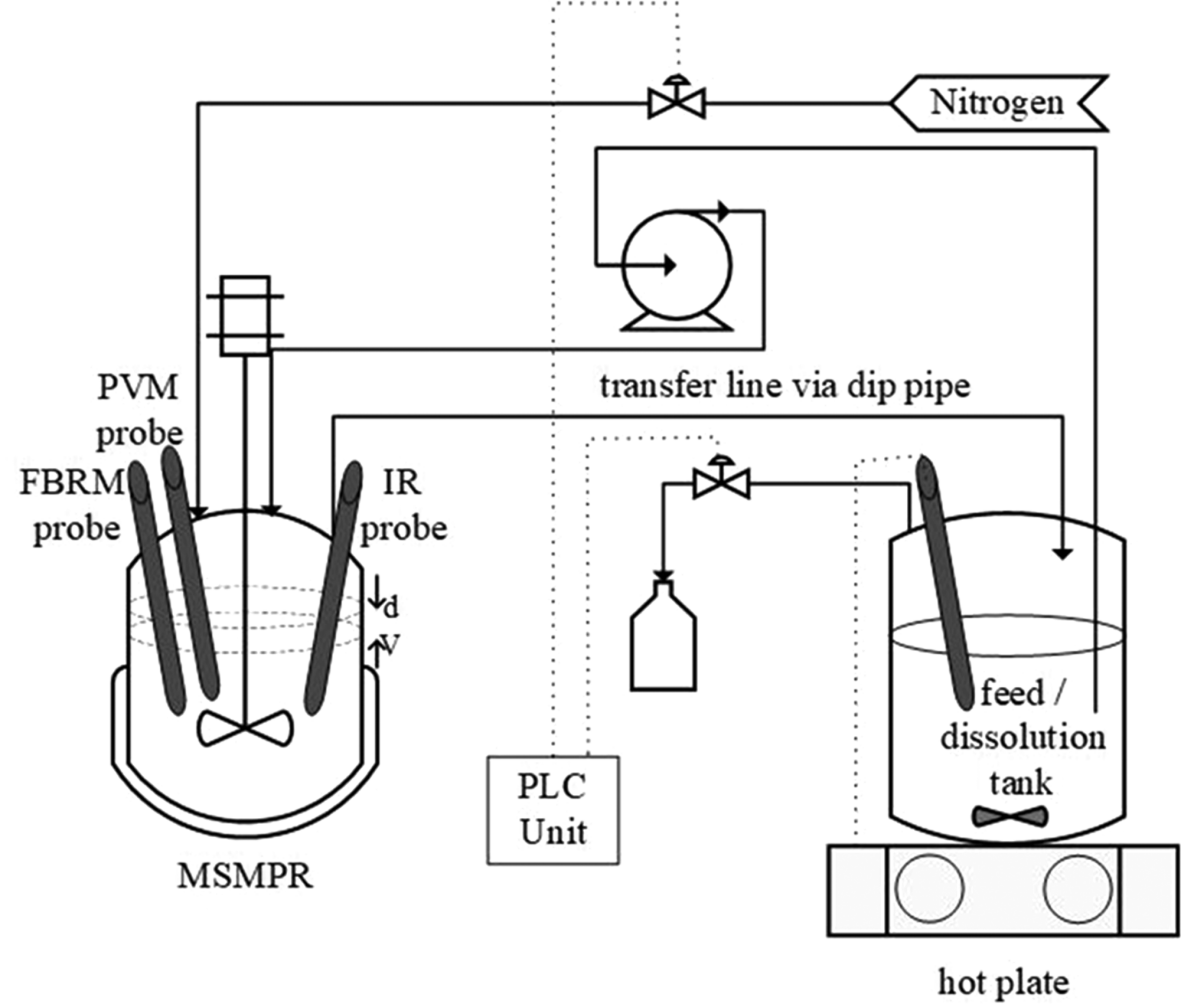
Schematic diagram of the MSMPR crystallizer experimental setup showing the process configured to operate in the product recycle mode.

**Figure 4:**
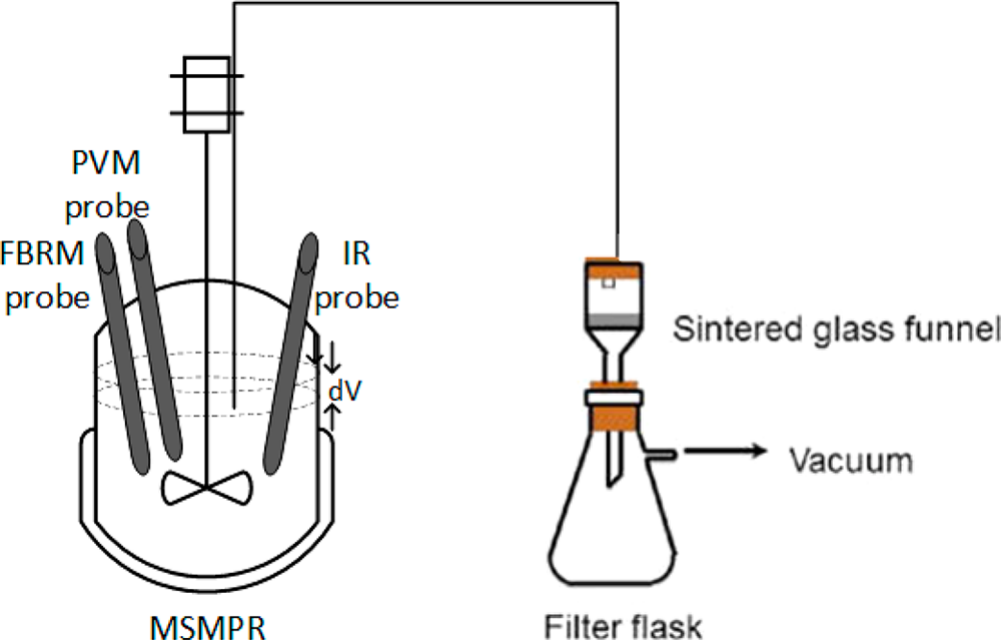
Schematic diagram of the equipment setup used to extract the product suspension from the MSMPR crystallizer.

The same reactor setup, a single stage mixed suspension mixed product removal (MSMPR) crystallizer with a feed and dissolution tank (Figure 3), was used for the MSMPR crystallization. A recycle system was employed using a 1 L Duran vessel with an independent temperature control, which was used as both the feed and product dissolution tank. The recycle system reduced the waste significantly during the MSMPR study, which was useful as a limited amount of product was available. Blocked transfer lines can be a challenge for the implementation of continuous crystallization at the lab scale.^32^ This was avoided by applying a rapid intermittent withdrawal of the slurry via a dip pipe.^14,15,28^ An automated nitrogen supply pressure source (50 kPa) was employed through the automation of a set of solenoid valves to transfer the product suspension from the crystallizer to the feed and dissolution tank. A programmable logic controller (PLC) unit (Siemens 230RCE) was used for this purpose. The amount of the product suspension transferred did not exceed 10% of the MSMPR content, and the time intervals for the withdrawal were calculated as the ratio of the transfer volume to the flow rate into the MSMPR crystallizer. The sampling of the product crystals from the MSMPR crystallizer was performed using the apparatus setup outlined in Figure 4. The product withdrawal pipe was redirected to a sealed and sintered glass funnel to capture the suspension sample instead of transferring the product to the feed tank. The filter cake was then washed with 50 mL of cold cyclohexane, collected, completely dried in a ventilated laboratory fume hood (at room temperature), and used for offline testing, such as SEM analysis.

### 2.3 Concentration Measurement

The gravimetric analysis method was used to measure the dissolved concentration in the crystallizer for both batch and continuous crystallizations. Using a glass pipet, approximately 1 mL of the crystalline suspension was extracted from the crystallizer and transferred into a 2 mL syringe. A syringe filter (PTFE, pore size of 0.2 μm) was used to filter the solids present in the suspension, and the clear liquor was transferred into a previously weighed glass vial. The glass vial with the clear liquor solution was put in an oven at 40 °C under vacuum (500 mbar) for 24 h to allow the solvent to evaporate. The mass of the sample was measured before and after drying using an electronic analytical balance (type LA124i, VWR, uncertainty of ±0.3 mg). The concentration was determined as
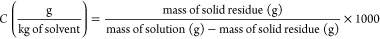
1Based on the dissolved concentration at 0 °C, the yield after the crystallization process could be calculated as
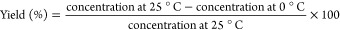
2where the concentration at 25 °C was obtained from the solubility curve of CNMP in toluene. For batch experiments, the dissolved concentration was measured at 0 °C after a 1 h hold period following cooling. For continuous experiments, the dissolved concentration was measured approximately every residence time during the MSMPR runs. As the product suspension was collected from the reactor at 0 °C, the glass pipettes, syringes, and syringe filters were all kept in the fridge to ensure a representative sample was measured.

### 2.4 Procedure for Agitation and Cooling Rate Variation

Nine different batch experiments were performed using different combinations of agitation and cooling rates, as outlined in Table 1, and each experiment was performed in triplicate. All other aspects of each experiment were carried out in the same manner. Initially, a saturated solution of CNMP in toluene at 25 °C was prepared and transferred to the crystallizer. The solution was heated to 30 °C and held at that temperature for 1 h to ensure full dissolution, which was confirmed by FBRM, ATR-FTIR, and PVM monitoring. The solution was then cooled to 0 °C at a fixed cooling rate as outlined in Table 1, followed by a 1 h hold at 0 °C under agitation to ensure the full de-supersaturation of the solution. The dissolved concentration was measured in triplicate using the gravimetric method, as described in Section 2.3. Afterward, the solution was heated back to 30 °C, and the outlined procedure was repeated at the same agitation and cooling rates on two further occasions. At the end of the third replicate, the process slurry was filtered. Solids were washed with 50 mL of cold cyclohexane, dried in air, collected, and used for any off-line analysis as required.

**Table 1:** Overview of Cooling and Agitation Rates Used in the Batch Experiment Runs

	cooling rates		
agitation (RPM)	1 K/min	0.5 K/min	0.1 K/min
250	√	√	√
425	√	√	√
600	√	√	√

### 2.5 Online Monitoring Using Process Analytical Techniques

While FBRM monitoring was performed throughout, specific points in the batch process were identified for comparison between different runs. The nucleation temperature was determined using the FBRM when an excess of 200 particles were detected in the size range of 0–1000 μm. The final chord length distribution (CLD) was obtained from the FBRM after a one-hour hold following the completion of the cooling step, and the average CLD of the three experimental replicates was taken. As the FBRM measures the number of particles that come into the probe scanning region per unit of time, the agitation rate can influence the CLD data as it increases the velocity of the solids flowing through the scanning region. A study performed by Dave et al.,^33^ showed that the increase of the agitation rates progressively increased the total number of counts, up to a plateau value. Therefore, to eliminate the influence of agitation rate on the CLD during different experimental conditions, batch experiments performed at 425 and 600 rpm, for example, a final CLD was taken after decreasing agitation rate to 250 rpm following the one-hour hold. The CLD can be presented as either unweighted or square-weighted; the unweighted CLD enhances the resolution of the changes to fine particles, while the square-weighted CLD enhances the resolution to coarse particles. PVM provides microscopy-quality images of the crystals in real-time throughout the process from the point of nucleation.

ATR-FTIR also provides information on the point at which nucleation occurs, with a sharp decrease in characteristic peak heights indicating a decrease in the dissolved concentration as material crystallizes out of solution. The characteristic peak height for CNMP was found to be 1700 cm^–1^ (Figure 4 of the SI). As the solvent can have a strong influence on the spectrum of a solution, the “subtract spectrum” feature on the iC IR software was utilized to subtract the toluene spectrum from the solution spectrum to enable the better visualization of the characteristic peaks for *p*-toluidine, α-chloropropionyl chloride, and CNMP. Additionally, 1700 cm^–1^ was identified as the characteristic peak to monitor for CNMP, which did not overlap with any peaks for any reagents used upstream to produce either CNMP or toluene.

### 2.6 Procedure for the Continuous Crystallization of CNMP

A saturated solution of CNMP in toluene at 25 °C was prepared with a concentration of 37.87 g/kg of toluene. The solution was transferred to the OptiMax reactor and the feed and dissolution tank. The MSMPR crystallizer was operated at an average operating volume of 300 mL where the dip pipe tube was placed, and 30 mL of the product suspension was withdrawn (Δ*V*) and returned to the feed and dissolution tank using rapid intermittent withdrawal. The volumetric flow rate (mL/min) used to transfer the feed into the MSMPR is listed in Table 2 and was calculated as
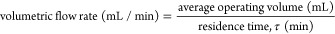
3The automated nitrogen supply pressure source (50 kPa) that was used to transfer the product suspension from the crystallizer to the feed and dissolution tank was controlled by a set of solenoid valves using a programmable logic controller (PLC) unit (Siemens 230RCE). The solenoid valve cycle time is listed in Table 2 and was calculated as
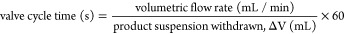
4The feed and dissolution tank was maintained at 35 °C to ensure the complete dissolution of the returning product crystals during system recycle mode. Prior to the start of the continuous MSMPR crystallization, a batch start-up was needed to produce the initial suspension for the MSMPR crystallizer. Following batch crystallization studies (Section 3.1), two batch start-up strategies were selected to investigate if the batch start-up strategy influences the time it takes for the MSMPR to reach a state of control. Experiments were performed with residence times (τ) of 20 min, 30 min, and 3 h and with agitation rates of 250 and 600 rpm to determine if any of these parameters influenced the steady state yield from the MSMPR. A total of nine experimental runs were performed, as listed in Table 2. FBRM was used to determine the onset of the steady state and to characterize the effect of the operating conditions on the particle size. FTIR was used to determine the onset of the steady state by tracking the mother liquor concentration throughout the MSMPR run. Offline concentration measurements were performed using gravimetric methods (as described in Section 2.3), complementing data obtained from the FTIR measurements. Finally, the crystal habit was monitored in situ throughout the crystallization study using PVM, which provides microscopy quality images of the crystals in process and in real time. All experiments are performed in triplicate. Average results of the triplicate runs are presented with error bars corresponding to the standard deviation.

**Table 2:** Process Conditions Used for the MSMPR Study

batch start-up strategy				MSMPR parameters		
exp no.	cooling rate (K/min)	agitation rate (RPM)	τ	agitation rate (RPM)	volumetric flow rate (mL/min)	valve cycle time (s)
1	1	250	20 min	250	15	120
2	0.5	600	20 min	250	15	120
3	0.5	600	20 min	600	15	120
4	0.5	600	20 min	600	15	120
5	1	250	30 min	600	10	180
6	0.5	600	30 min	600	10	180
7	0.5	600	30 min	600	10	180
8	1	250	3 h	250	1.67	1080
9	1	600	3 h	600	1.67	1080

## 3 Results and Discussion

### 3.1 Characterization of the Batch Crystallization for the Start-Up Investigation

**Figure 5:**
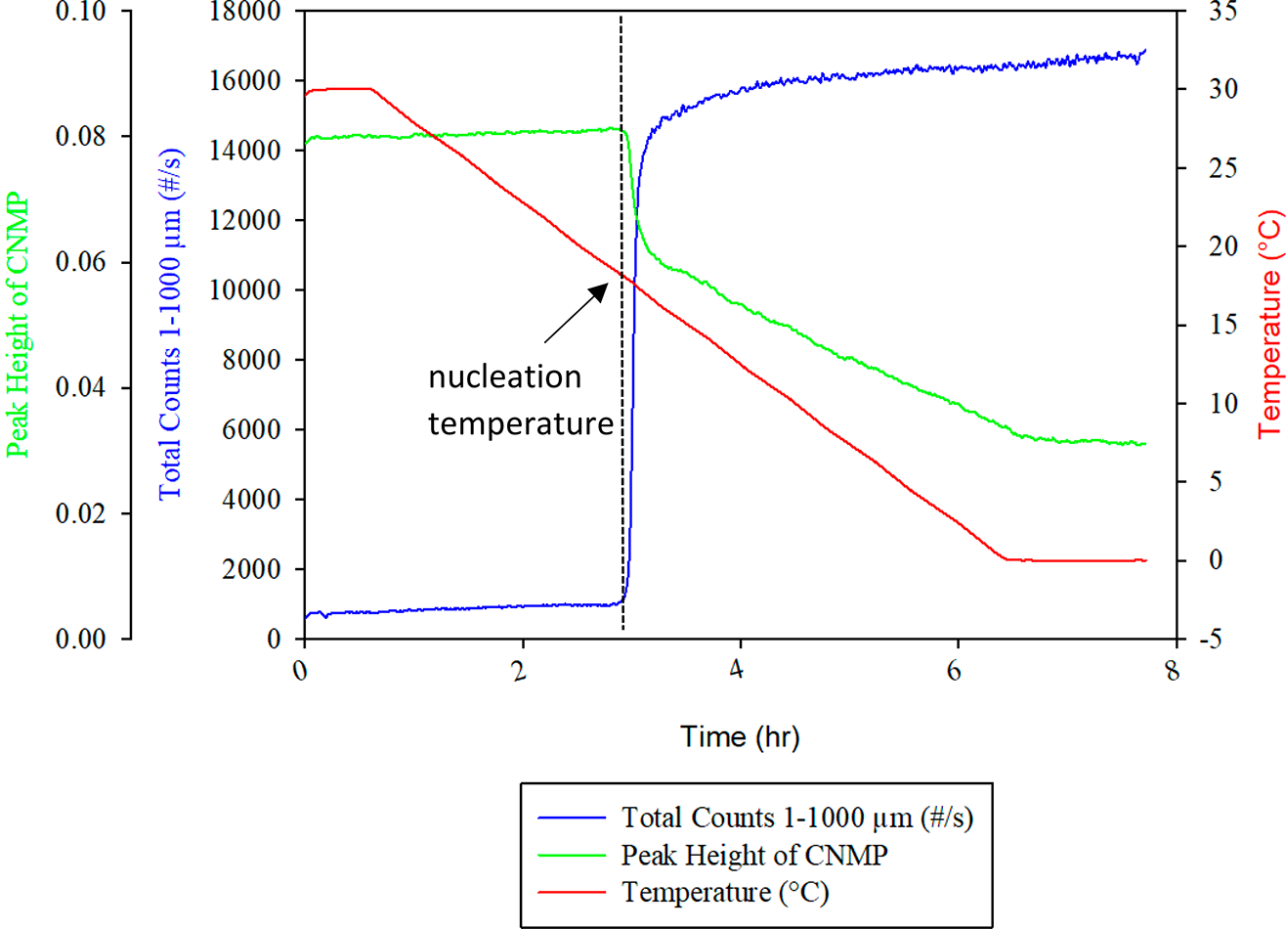
FBRM and FTIR trends for an experimental run with an agitation rate of 600 rpm and a cooling rate of 0.1 K/min.

The nucleation temperature for each experimental run was determined using FBRM and FTIR (Figure 5). The onset of nucleation was determined where a sudden increase in the number of particles was observed in the size range of 0–1000 μm (by FBRM). FTIR confirmed this point of nucleation via a sharp decrease in the characteristic peak height corresponding to CNMP, indicating a decrease in the dissolved concentration as materials came out of solution.

**Figure 6:**
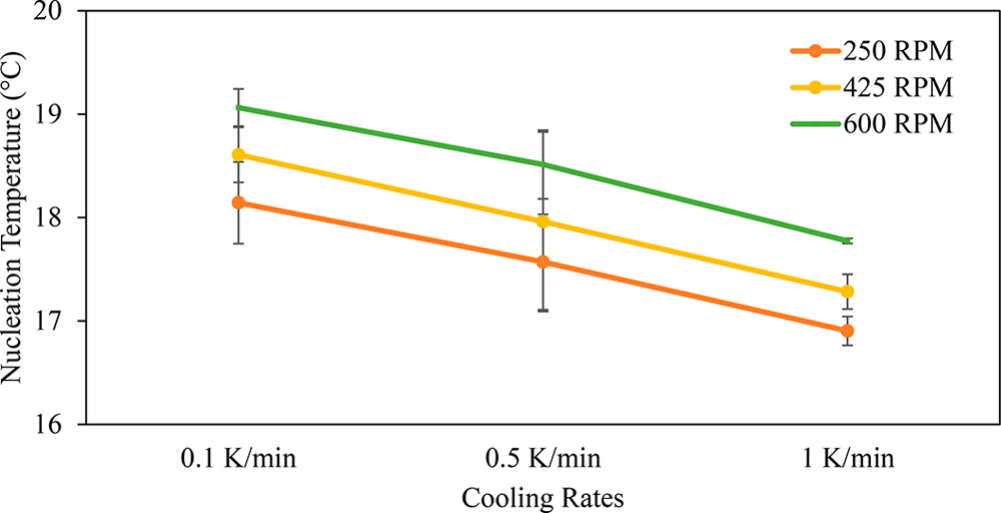
Nucleation temperature at various agitation and cooling rates. The standard deviation error bars are shown.

Average nucleation temperatures for different conditions are presented in Figure 6, which indicates that an increase in the cooling rate leads to a decrease in the nucleation temperature. This result was expected, as slower cooling rates have a narrower metastable zone width (MZW) while faster cooling rates have a wider MZW. Increasing the agitation rate leads to a corresponding increase in the nucleation temperature (decrease in the MZW). This was another expected result, as an increased agitation rate can increase the rate of secondary nucleation.

**Figure 7:**
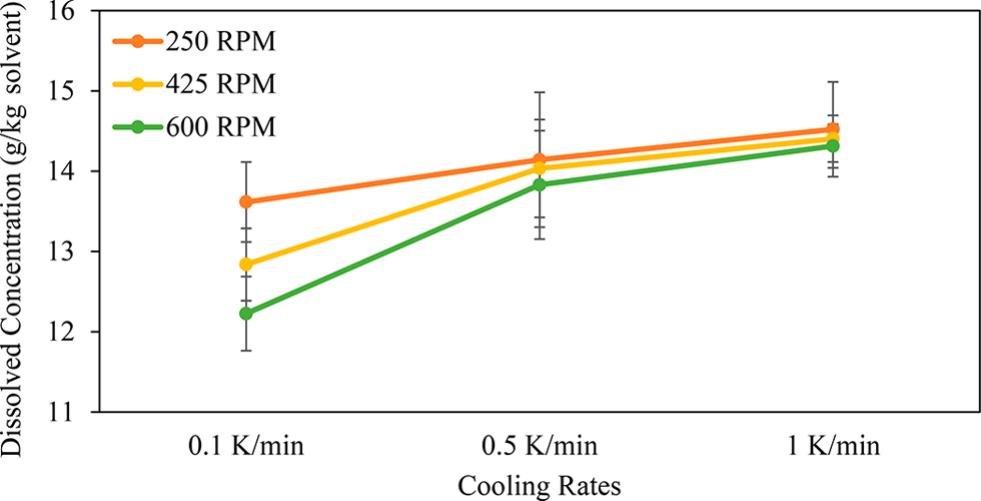
Dissolved concentration at 0 °C obtained after a 1 h hold for various agitation and cooling rates. The standard deviation error bar are shown.

The average dissolved concentration at the end of a one-hour hold following cooling is presented in Figure 7. Using these average dissolved concentration values, the average yield (%) of the batch crystallization after the one-hour hold was determined, and the data are presented in Table 3. A decrease in the cooling rate resulted in a decrease in the value of the dissolved concentration at 0 °C after the one-hour hold, leading to an increase in the yield. Slower cooling rates allow for the supersaturated solution to nucleate for a longer period; it was expected that a slower cooling rate would provide a higher yield. However, increasing the agitation rate decreases the value of the dissolved concentration at 0 °C after the one-hour hold, leading to an increase in the yield. As discussed in relation to the nucleation temperature, an increase in the agitation rate can affect the secondary nucleation in the system, which leads to an increased yield. Furthermore, it can be seen from Figure 7 that at slower cooling rates the effect of the agitation rate is more significant compared to that at faster cooling rates. The results show that the agitation rate of 600 rpm with a cooling rate of 0.1 K/min gives the highest yield out of the nine experiments. To maximize the yield, the agitation rate should be maximized and the cooling rate should be minimized.

**Table 3:** Average Yield (%) after a One Hour Hold during Batch Studies at Various Agitation and Cooling Rates

	0.1 K/min	0.5 K/min	1 K/min
250 rpm	64.05	62.65	61.65
425 rpm	66.10	62.94	61.96
600 rpm	67.72	63.48	62.20

### 3.2 Characterization of MSMPR Crystallization: Identifying the Steady-State Operation of the MSMPR Crystallization

**Figure 8:**
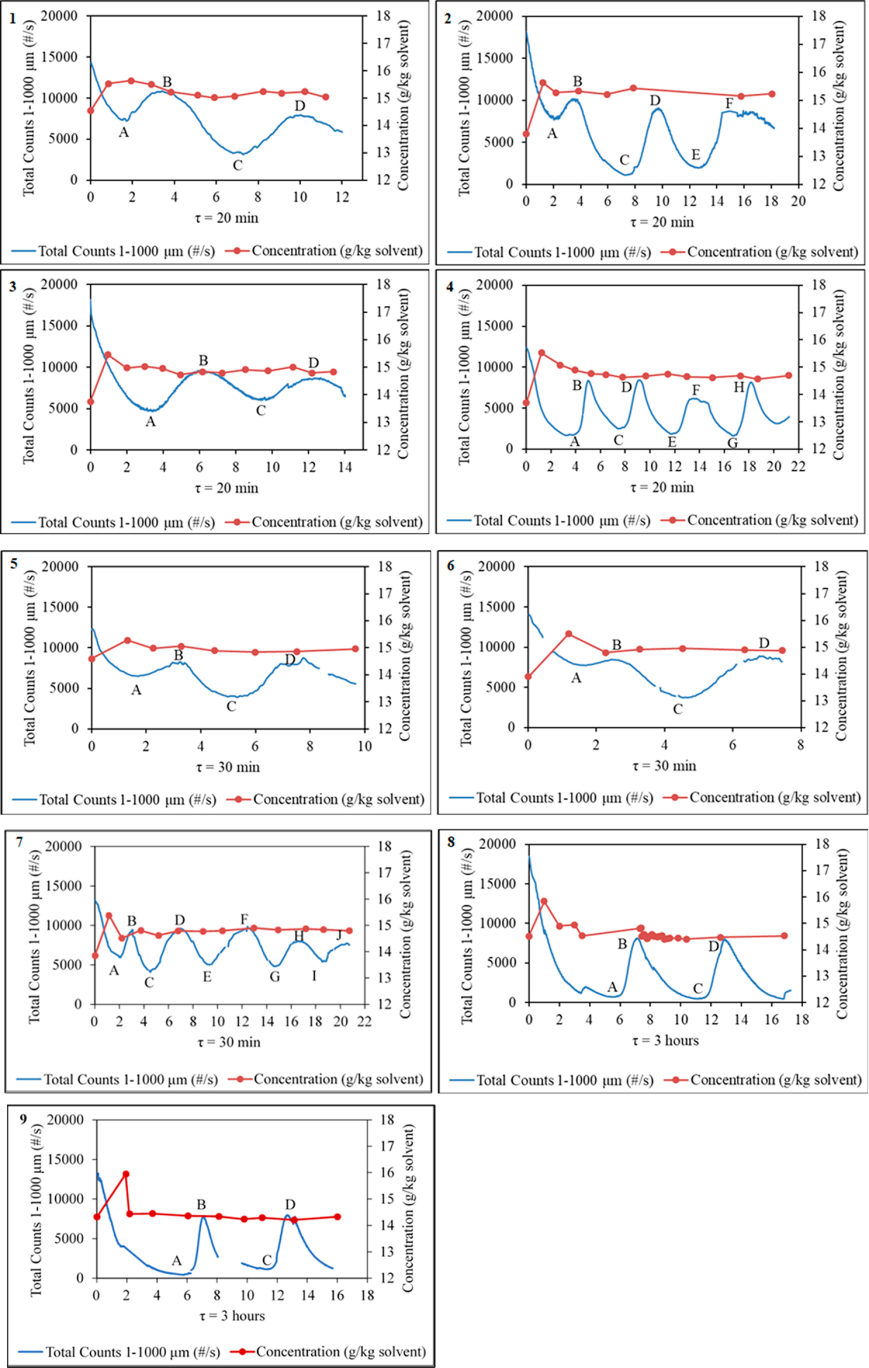
Change in FBRM total counts and the mother liquor concentration (g/kg of solvent) during MSMPR crystallization for experiments 1–9.

Figures 8 shows the change in FBRM total counts and the mother liquor concentrations versus time for experiments 1–9 (Table 2). The total counts oscillate and do not reach the expected steady state where they are constant with time; instead, they reach a state of control with regular oscillations of the total counts. MSMPR crystallization experiments were performed for longer to see if the oscillation in the change of the FBRM total counts would gradually decrease. Figure 8 shows that the observed oscillation did not disappear despite increasing the run time of the experiment, with the size of the oscillation remaining constant after 21 residence times.

Initially, as the MSMPR operation began, an increase in the concentration was observed, which stabilized after approximately two residence times. This stabilization is reflected in the FTIR versus time data for experiments 1–7 (Figure S5 in the SI). The FTIR trends initially increase and then subsequently stabilize, supporting the concentration data obtained offline (FTIR data for τ = 3 h (experiments 8 and 9) are unavailable due to severe fouling of the FTIR over long periods of time).

In PVM images collected at the peaks and troughs of the experimental runs (Figures S6 and S7 in the SI) for residence times of 20 and 30 min, the morphology of the crystals at the peaks is thin needles, while images at the troughs show a wider needle-shaped crystal. For the residence time of 3 h, the morphology of the crystals at the peaks is needles, as observed in the shorter residence times, while images collected at the troughs show a platelet morphology. For experiment 8, a solid sample was taken at the trough and the peak, as indicated in Figure S8. XRD analysis of the samples was performed to determine whether the two morphologies were a result of different polymorphs. However, the two samples had the same XRD pattern. Therefore, it can be concluded that the plate- and needle-shaped crystals are not polymorphs of CNMP.

MSMPR crystallizers can cause cyclical oscillations in the crystal size distribution (CSD),^34^ which was seen in this case. It has been reported in the literature that cycles in the particle size were observed in the continuous crystallization of potassium chloride using MSMPR.^35^ Analytical studies show there are two types of CSD instability, namely high-order and low-order cycling. In the study of potassium chloride, low-order cycling applies to the system, as the oscillation is caused mainly by classified product removal and aggravated further by the destruction of fines and clear liquor advance.^35^ Classified product removal did not occur in the current MSMPR setup, as intermittent withdrawal, which has been widely studied in the literature,^14−16,28^ was implemented for all experimental runs to avoid the classification of crystals during product removal. Furthermore, the oscillations do not correlate in any way with the removal rate. The other type of CSD instability typically observed is high-order cycling. High-order cycling may be the case in the system described here, as it is highly dependent on the form of the nucleation kinetics and occurs with a high-order relationship of nucleation to supersaturation, such as nucleation discontinuity.

### 3.3 Investigating the Impact of the Batch Start-Up Strategy on the Time to Steady State

The goals of the batch studies was to better understand if the cooling and agitation rates affect the final CLD and, in turn, to investigate the impact of the initial start-up CLD (following the batch start-up crystallization) effects on the time required for the MSMPR crystallizer to reach a state of control. The ideal batch start-up strategy is one that requires the least time to achieve a state of control (steady-state operation). To achieve this, a comparison between the start-up strategies of the two batch crystallizations was performed based on which produced the smallest or largest material. Ultimately, the strategy with the shortest time to the steady state should be chosen for the optimized process.

**Figure 9:**
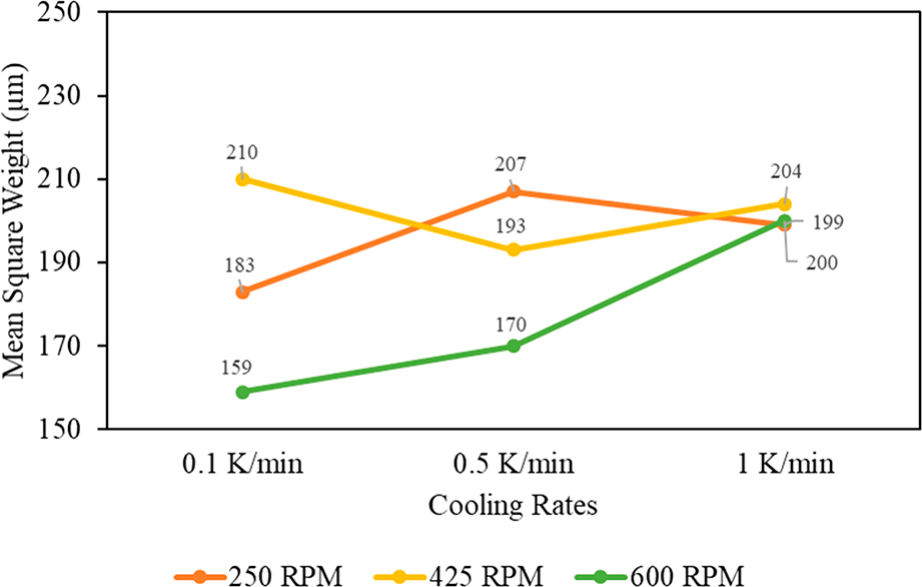
Mean-square weights (μm) vs cooling rates at various agitation rates.

**Figure 10:**
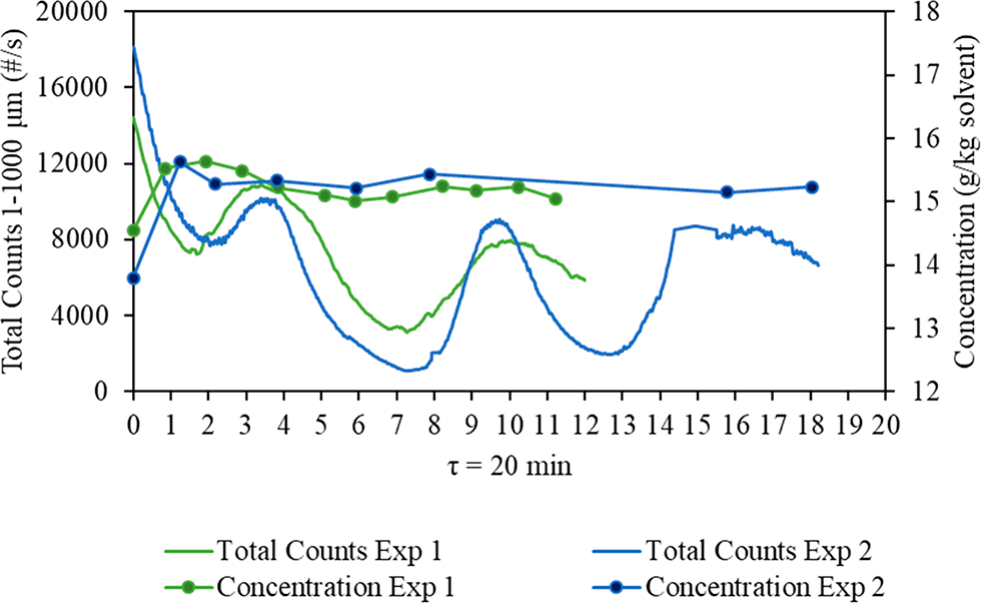
Comparison between two batch start-up strategies: 1 K/min and 250 rpm for experiment 1 versus 0.5 K/min and 600 rpm for experiment 2 (20 min residence time).

**Figure 11:**
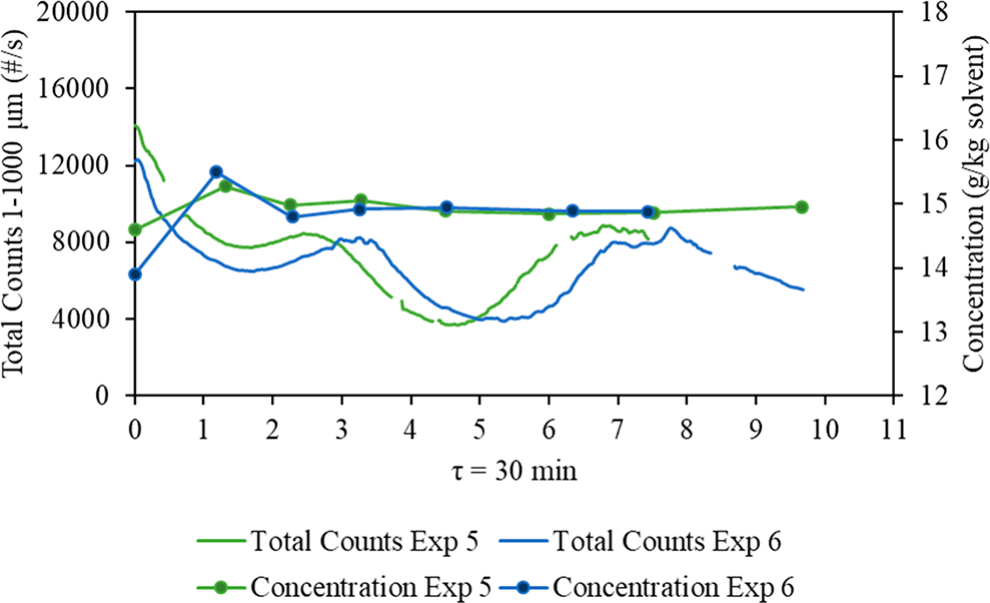
Comparison between two batch start-up strategies: 1 K/min and 250 rpm for experiment 5 versus 0.5 K/min and 600 rpm for experiment 6 (30 min residence time).

Figure 9 shows the final FBRM mean-square weight for each of the different batch experiments. The mean-square weight is similar for different cooling rates at agitations of 425 and 250 rpm. For 425 rpm, there is less than a 10% change with the cooling rate. At 600 rpm, there is a clear increase in the mean-square weight with the cooling rate. It is useful in this case to also examine the images of the final particles produced in each run (the SEM images (Figure S3) and PVM images (Figure S4 are included in the Supporting Information). A cooling rate of 0.1 K/min and an agitation rate of 600 rpm provided the lowest mean-square weight of 159 μm (Figure 9). The batch start-up parameters that gave the next smallest particle size were 0.5 K/min and 600 rpm, producing a mean-square weight of 170 μm; these parameters were chosen as one batch start-up strategy. The cooling rate of 0.1 K/min was not chosen as a batch start-up strategy as it would be inefficient to perform due to the 5 h process time for cooling from 30 to 0 °C. The difference of 11 μm did not warrant the much longer run time. This decision was supported by an examination of the PVM images of the final particles (Figure S4). The final particles for both runs are similar. A batch crystallization that produced the larger crystals was chosen for direct comparison. The batch start-up with 1 K/min as the cooling rate and 250 rpm agitation rate was selected. This selection was based on an examination of the crystal images (Figures S3 and S4) and the further examination of the FBRM data. These start-up strategies were compared for two different residence times of 20 (Figure 10) and 30 min (Figure 11).

As previously discussed, the FBRM total counts oscillate in this system. For the two chosen batch start-up strategies, the observed oscillations were similar, and the time to the steady state with respect to the dissolved concentration was the same and did not appear to affect the onset of oscillation of the FBRM counts. Consequently, it can be concluded that the batch start-up does not influence time to the steady state for the MSMPR operation in the case of this specific system. It was decided to use the cooling rate of 1 K/min for the batch start-up for future operations as it takes the shortest operational time and will be the most efficient. The chosen agitation rate for the batch start-up will be the same as that chosen for the continuous MSMPR operation.

### 3.4 the Effect of the Agitation Rate on the Operation of the MSMPR System

**Figure 12:**
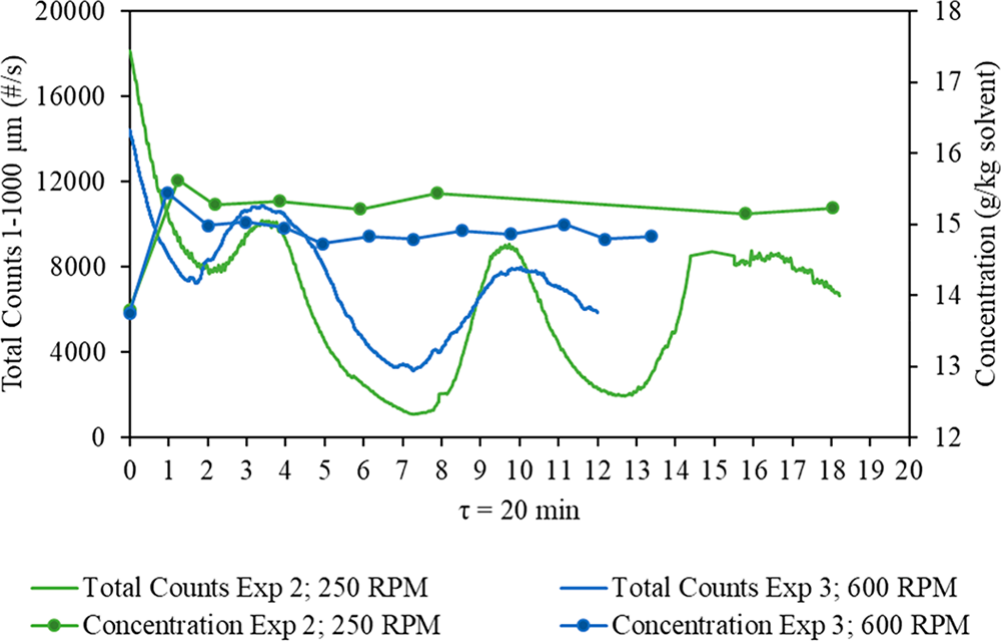
Comparison of total counts 1–1000 μm (number per second) and concentration (g/kg of solvent) between two agitation rates (250 rpm for experiment 2 and 600 rpm for experiment 3) at a residence time of 20 min.

**Figure 13:**
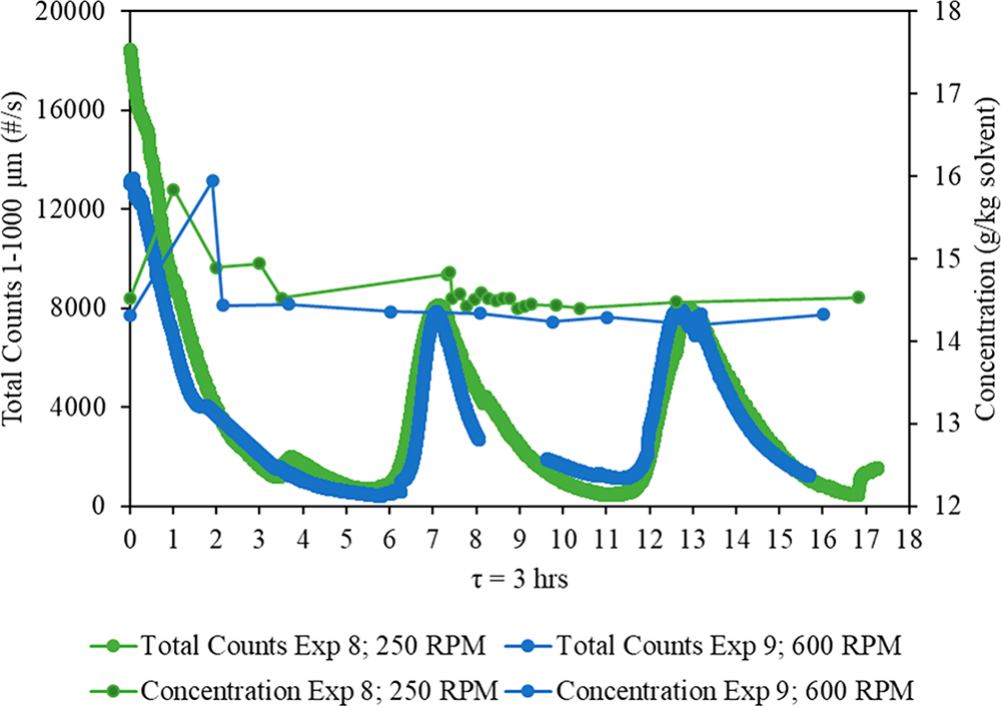
Comparison of total counts 1–1000 μm (number per second) and concentration (g/kg of solvent) between two agitation rates (250 rpm for experiment 8 and 600 rpm for experiment 9) at a residence time of 3 h.

The effect of low (250 rpm) and high (600 rpm) agitation rates on the continuous crystallization of CNMP in a MSMPR crystallizer was investigated. Experiments 2 and 3 with a residence time of 20 min were performed at 250 and 600 rpm, respectively, while experiments 8 and 9 with a residence time of 3 h were performed at 250 and 600 rpm, respectively. The FBRM trends showing the total counts 1–1000 μm (number per second) and the mother liquor concentration (g/kg of solvent) measured gravimetrically throughout the MSMPR run are presented in Figure 12 for τ = 20 min and Figure 13 for τ = 3 h. Similar oscillation patterns were observed for both agitation rates, and the steady state was reached after two residence times regardless of the agitation rate.

Table 4 shows the concentration at the steady state, *C* (g/kg of solvent), which is the average of the concentration taken from the point during the MSMPR run where the concentration is stable. Using these concentration values, the yield (%) of the MSMPR was calculated. The observed yield was slightly higher at 600 rpm compared to that obtained at 250 rpm. For τ = 20 min the yield increased from 58.91% to 59.96% between 250 and 600 rpm, respectively, while for τ = 3 h the yield increased from 60.82% to 61.44% between 250 and 600 rpm, respectively. The increase in agitation led to an increase in secondary nucleation, which resulted in a slightly higher yield. One of the major goals of this study was to develop a continuous process that produced the highest consistent yield of CNMP that could feed a secondary reaction via a solvent swap. In this regard, future continuous crystallizations of CNMP in a MSMPR crystallizer will be performed operationally at an agitation rate of 600 rpm and a residence time of 20 min.

**Table 4:** Comparison of the Average Concentration at the Steady State and the Yield for Agitation Rates at 250 and 600 rpm

		250 rpm	600 rpm
concentration (g per kilogram of solvent)	τ = 20 min	15.27	14.88
	τ = 3 h	14.56	14.33
yield (%)	τ = 20 min	58.91	59.96
	τ = 3 h	60.82	61.44

### 3.5 The Effect of Residence on the Operation of the MSMPR System

**Figure 14:**
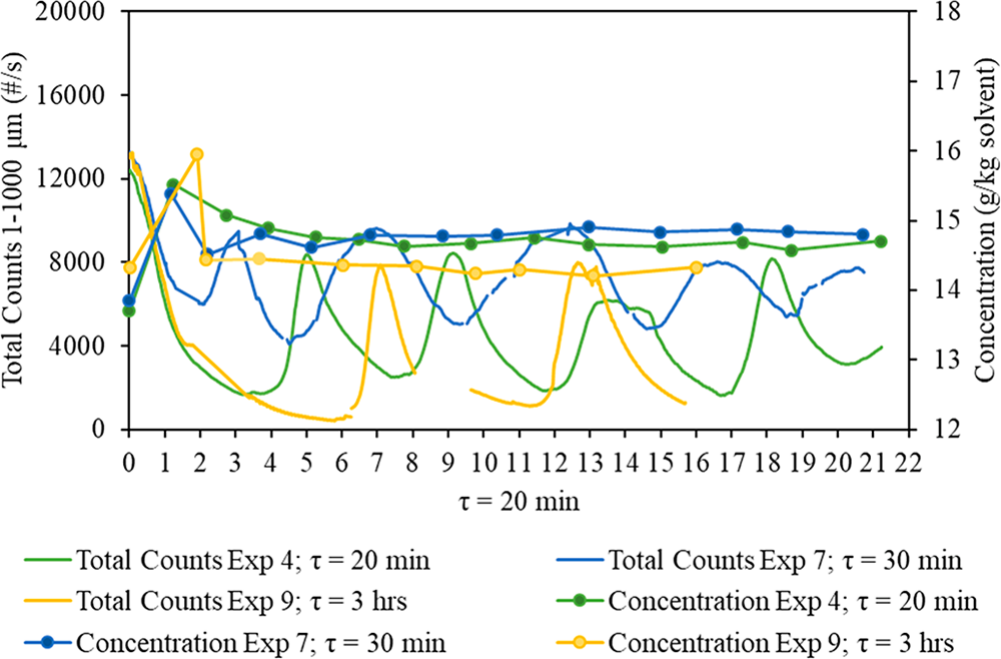
Comparison of total counts 1–1000 μm (number per second) and concentration (g/kg of solvent) among the following different residence times: 20 min for experiment 4, 30 min for experiment 7, and 3 h for experiment 9.

The effect of the residence time on the continuous crystallization of CNMP in a MSMPR crystallizer was examined by varying the residence time (20 min, 30 min, and 3 h). Figure 14 shows the FBRM total counts 1–1000 μm (number per second) and the mother liquor concentration (g/kg of solvent) versus time for experiments 4 (τ = 20 min), 7 (τ = 30 min), and 9 (τ = 3 h). The total counts oscillated, as discussed in previous sections, and the mother liquor concentration reached stability after two residence times for all the residence times investigated. Table 5 shows that the yield is similar between τ = 20 and 30 min and slightly higher for 3 h by ∼1%. Despite the slightly higher yield at 3 h, the overall productivity (mass of the product produced versus time for the same scale) would be higher at shorter residence times. Therefore, a residence time of 20 min will be used to perform the continuous crystallization of CNMP in a MSMPR crystallizer, along with an agitation rate of 600 rpm.

**Table 5:** Comparison of the Average Concentration at the Steady State, the Yield, and the Productivity for Residence Times of 20 min, 30 min, and 3 h

	exp 4 τ = 20 min	exp 7 τ = 30 min	exp 9 τ = 3 h
concentration (g/kg of solvent)	14.70	14.78	14.33
yield (%)	60.46	60.24	61.44
productivity (g/h)	69.51	46.18	7.85

## 4 Conclusion

The continuous crystallization of CNMP was performed successfully using a MSMPR system. The final optimized process parameters fit with the feed rate and the scale required to directly supply the system from a previous continuous flow chemistry step, which produced CNMP in solution. The FBRM count and CLD trends for the system, along with PVM and offline particle imaging, indicate that the PSD oscillates and reaches an unusual state of control. The instability of the CSD can be described as high-order cycling, which is highly dependent on the form of the nucleation kinetics and occurs with a high-order relationship between nucleation and supersaturation, such as nucleation discontinuity. Despite the oscillations, the mother liquor concentration for all the residence times reached stability after two residence times, which was supported by the FTIR data.

The batch study for the cooling crystallization of CNMP in toluene at various agitation and cooling rates was successfully investigated and characterized. The goal of the batch studies was to gain a better understanding of whether variations in the cooling rate and agitation rate affected the PSD during the batch start-up crystallization and if the crystal size produced during the batch start-up would influence the time it took for the MSMPR crystallizer to reach a state of control. Overall, the batch crystallization of CNMP was found to be very robust with respect to changes in cooling and agitation rates. Of the various conditions investigated, two batch start-up strategies were chosen for subsequent investigation, which resulted larger and smaller mean-square weights following batch crystallization. In both cases, the time to the steady state for the dissolved concentration was the same and did not appear to affect the onset of oscillation of the FBRM counts. Therefore, it can be concluded that the batch start-up does not influence the time to the steady state for the MSMPR operation in the case of this specific system. Conditions for the batch start-up can be selected based on providing the shortest process time to improve the efficiency of the system.

Increasing the agitation rate led to an increase in secondary nucleation, resulting in a slightly higher yield. Therefore, we recommend that the continuous crystallization of CNMP in a MSMPR crystallizer should be performed at the higher agitation rate of 600 rpm. Finally, the effect of the residence time on the system was determined by varying the residence time. The yield was similar for the three residence times selected for investigation, with a slight increase of 1% for 3 h. Despite the slightly higher yield at 3 h, the overall productivity was higher at shorter residence times, with 69.51 g/h for τ of 20 min versus 7.85 g/h for τ of 3 h. Therefore, a residence time of 20 min will be used to perform the continuous crystallization of CNMP in a MSMPR crystallizer.

Despite the oscillation that occurred in the FBRM total counts and the chord length distribution, the mother liquor concentration reached a state of control after two residence times, and a consistent solid form was produced. In this way, the system reaches a state of control with regular oscillations of the total counts but a constant yield. The overall goal of this study was to develop an intermediate crystallization step to produce the highest constant yield of pure CNMP. In future work, this crystallization step will be fed by a chemical reaction step and in turn continuously supply the pure CNMP intermediate for a subsequent reaction step. In preparation for the subsequent step, it will be redissolved in a “solvent swap” and forward processed to the next reaction step. While it would be ideal for the PSD to be consistent for the filtration and redissolving process step, in this case it is not essential for the overall process requirements. The priority is to develop a continuous process that produces the highest consistent yield of CNMP, which was achieved by operating the MSMPR crystallizer at an agitation rate of 600 rpm and a residence time of 20 min.

## Supporting Information Available

NMR spectra, FTIR spectra, SEM image of crystal sizes, PVM images of crystal sizes, FTIR trends during crystallization, PVM images the crystal morphology during crystallization, and XRD analysis (PDF)

## Abreviations

ATR-FTIR: attenuated total reflectance Fourier transform infrared spectroscopy
C: concentration (g/kg of solvent)
CLD: chord length distribution
CNMP: 2-chloro-*N*-(4-methylphenyl)propanamide
CSD: crystal size distribution
FBRM: focused beam reflectance measurement
MSMPR: mixed suspension–mixed product removal
MZW: metastable zone width
PAT: process analytical technology
PVM: particle vision and measurement
PFC: plug flow crystallizer
STD: standard deviation
T_N_: nucleation temperature
τ: residence time
Δ*V*: product suspension withdrawn
