# Supplementary material for: Design and Optimization of the Single-Stage Continuous Mixed Suspension–Mixed Product Removal Crystallization of 2-Chloro-N-(4-methylphenyl)propenamide
Source: ACS Omega. 2022 Apr 13;7(16):13676–86. doi: 10.1021/acsomega.1c07228 (PMC9088942; doi:10.1021/acsomega.1c07228)
Supplement: Supplementary file 1 — ao1c07228_si_001.pdf [file ao1c07228_si_001.pdf]

Design and optimization of a single stage continuous MSMPR crystallization of 2-Chloro-N-(4-methylphenyl)propenamide.

Gladys Kate Pascual[a], Philip Donnellan[a], Brian Glennon[a,b], Barbara Wood[a,b]* and Roderick C Jones[a]*

[a]*Synthesis and Solid State Pharmaceutical Centre (SSPC), School of Chemical and Bioprocess Engineering, University College Dublin, Belfield, Dublin 4, Ireland*

[b]*APC Ltd, Cherrywood Business Park, Loughlinstown, Co Dublin, Ireland*

*Corresponding Authors

Corresponding author emails: roderick.jones@ucd.ie, barbara.wood@approcess.com

Table of Contents:

General Procedures..P2

[1]NMR spectra of 2-chloro-N-(p-tolyl)propenamide……………………………………………………….P2

FTIR spectrum of CNMP in Toluene………………………………………………………………………………P3

SEM image of various cooling and agitation rates on crystal size……………………………………….P3

PVM images of various cooling and agitation rates on crystal size……………………………………P4

FTIR trend throughout the MSMPR crystallization……………………………………………………………P5

PVM images on the peaks and troughs throughout the MSMPR crystallization………………….P6

XRD analysis for Sample A obtained at the trough and Sample B……………………………………..P8

References………P8

General Procedures. All solvents and reagents were commercially obtained from Sigma Aldrich and used as received without further purification (Table 1). ^{1}H, ^{13}C, NMR spectra were recorded in $CDCl_3$ were recorded on a 300 and 400 MHz instrument. Chemical shifts (δ) are reported in parts per million (ppm) and are referenced to the residual solvent peak. The order of citation in parentheses is (1) multiplicity (s = singlet, d = doublet, t = triplet, q = quartet, m = multiplet, b = broad), (2) coupling constant (J) quoted in hertz to the nearest 0.1 Hz, and (3) number of equivalent nuclei (by integration). EI HRMS measurements were taken on TOF mass analyser.

Synthesis of 2-chloro-N-(p-tolyl)propanamide, (CNMP)

2-chloropropionyl chloride (0.78 mL, 7.98 mmol, 1.2 equiv.) was added dropwise (with caution) at 0°C, to a rapidly stirred biphasic mixture of *p*-toluidine (0.71 g, 6.65 mmol, 1 equiv.) in toluene (40 mL) and aqueous sodium hydroxide (0.4 g, 9.98 mmol, 1.5 equiv. in 40 mL of deionised water). Once the addition was complete, the mixture was warmed to room temperature and stirred for a further 1 hr and the organic layer was extracted with toluene (3 x 10 mL). The organic fractions were combined, dried over $MgSO_4$, filtered and the solvent removed *in vacuo* resulting in an off-white solid (1.29 g, 99 %). Spectral data of the obtained product matched that reported in the literature[1,2].

^{1}HNMR (300 MHz, $CDCl_3$): δ 8.21 (s, 1H), 7.42 (d, *J* = 8.2 Hz, 2H), 7.15 (d, *J* = 8.2 Hz, 2H), 4.54 (q, *J* = 7.1 Hz, 1H) 2.13 (s, 3H), 1.83 (d, *J* = 7.1 Hz, 3H). ^{13}C NMR (100 MHz, $CDCl_3$): δ 166.9, 134.4, 134.0, 129.1, 119.7, 55.9, 22.4, 20.5. HRMS (EI) *m/z* $[M]^+$: found 197.0604 $[C_{10}H_{12}ClNO]^+$, calcd 197.0607.

Figure S1. 1H NMR spectrum of 2-chloro-N-(4-methylphenyl)propenamide (CNMP) in CDCl3 at 300 MHz

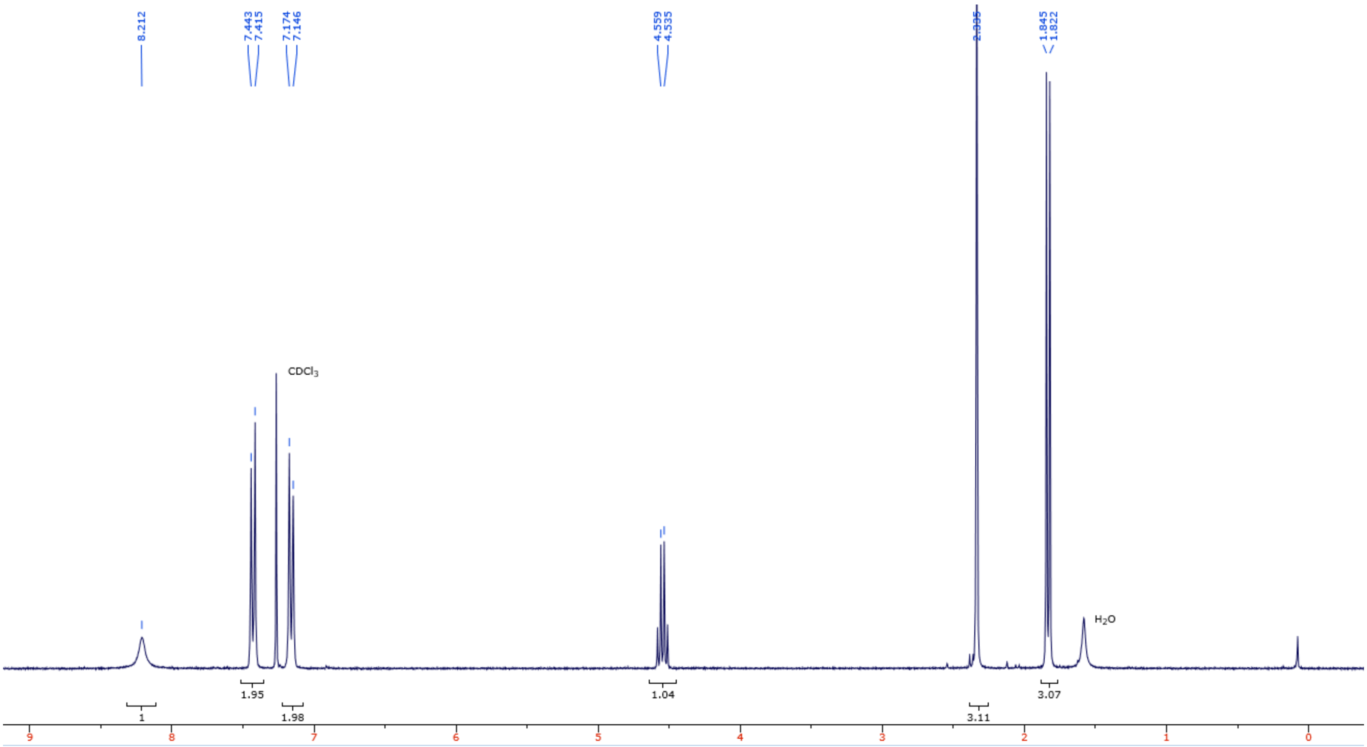

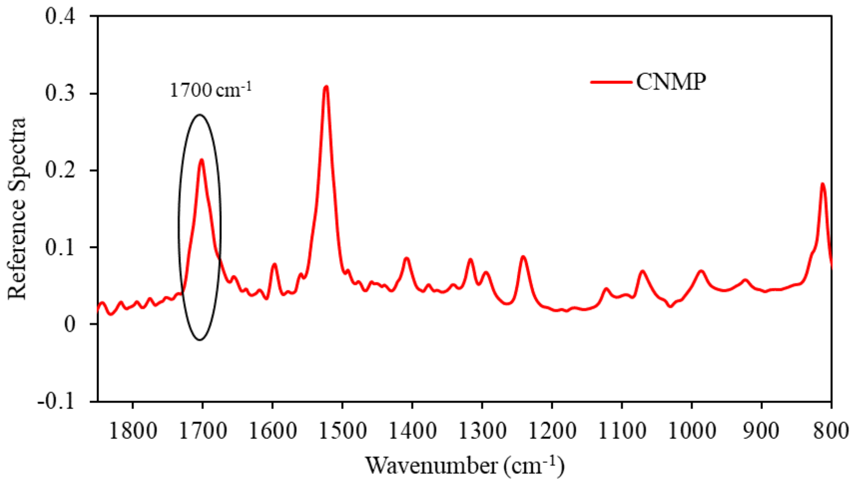

Figure S2. Section of the FTIR spectrum of CNMP/Toluene solution at 60 °C with the toluene spectrum subtracted. The characteristic (C=O) peak for CNMP indicated at 1700 cm^{-1}.

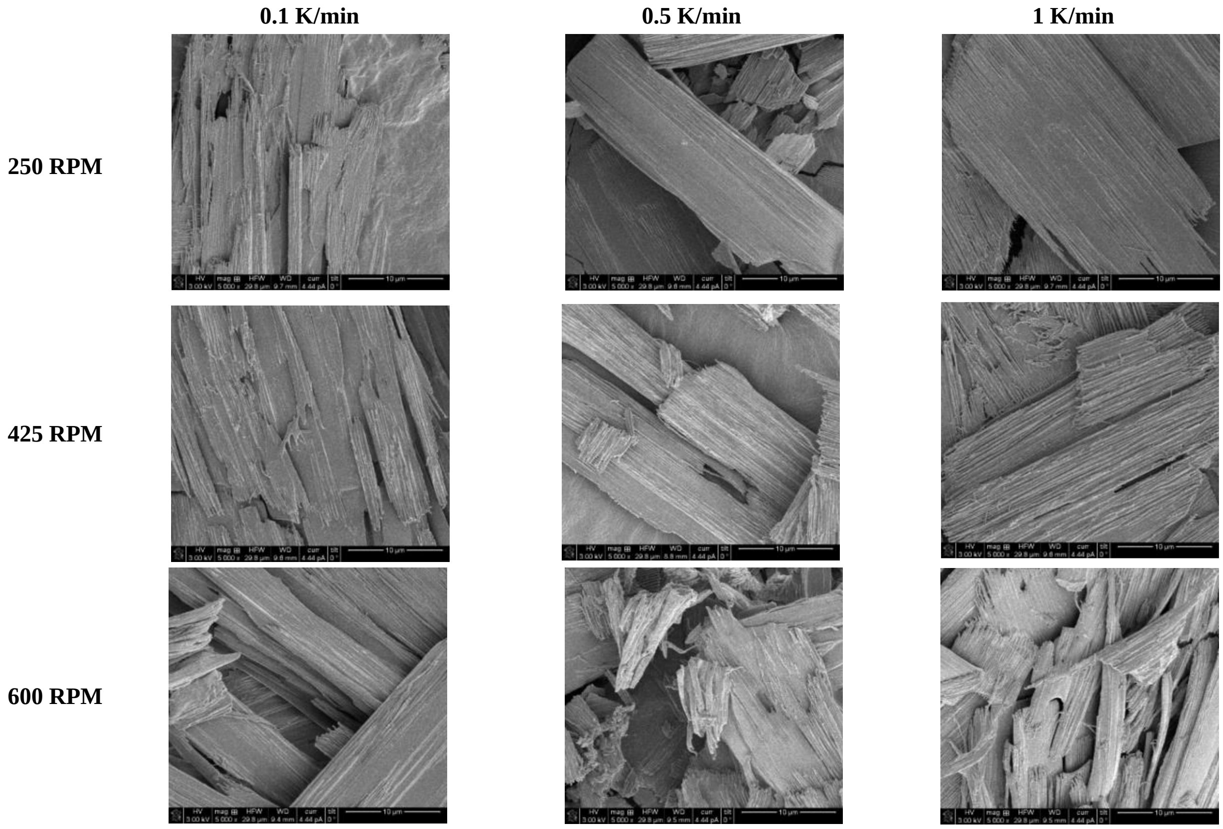

Figure S3. SEM images of CNMP at magnification of 5000, at various cooling and agitation rates.

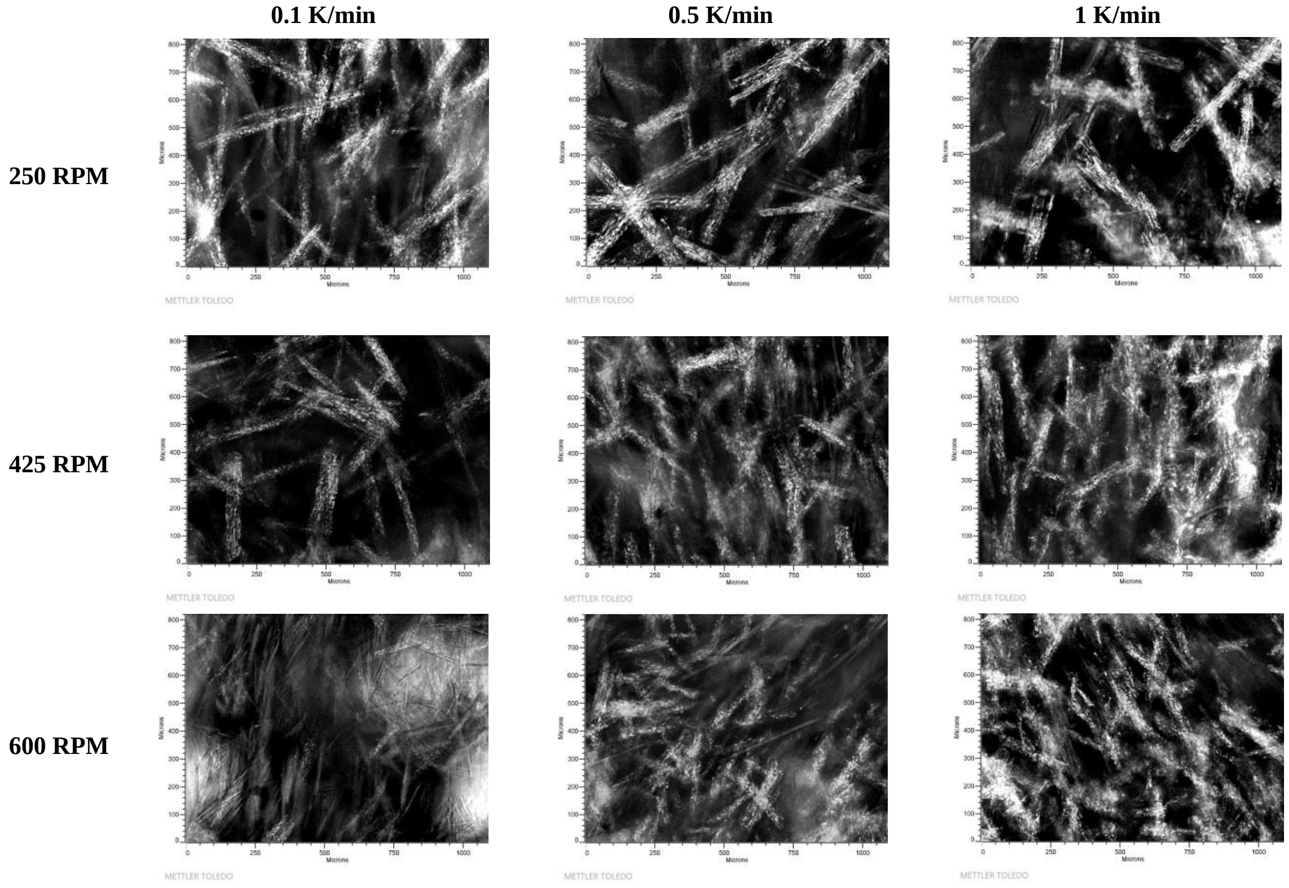

Figure S4. PVM images of CNMP obtained after one hour hold for each experiment of varying cooling and agitation rate.

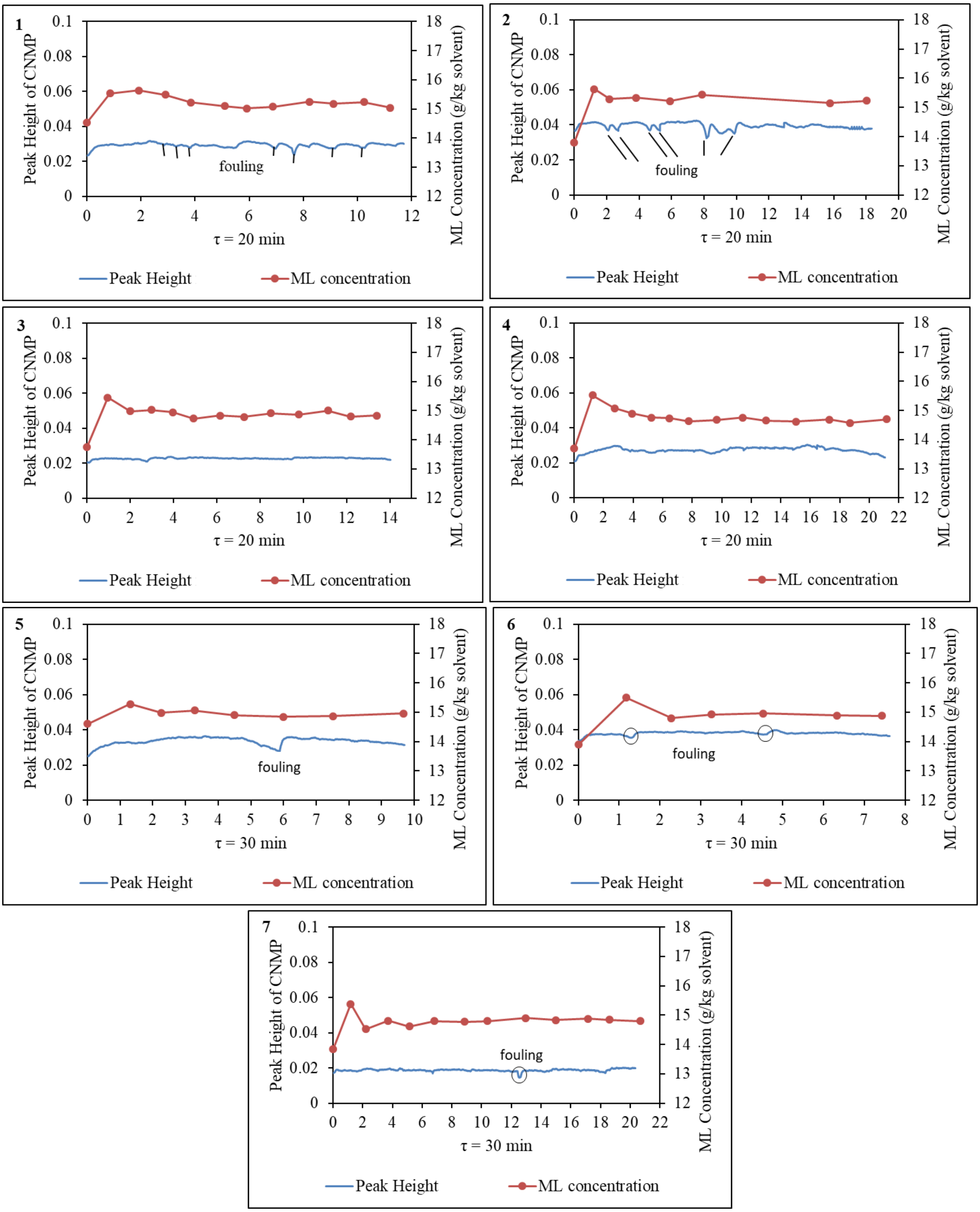

Figure S5. Change in mother liquor concertation (g/kg solvent) overlapped with FTIR trend throughout the MSMPR crystallization for exp 1-7

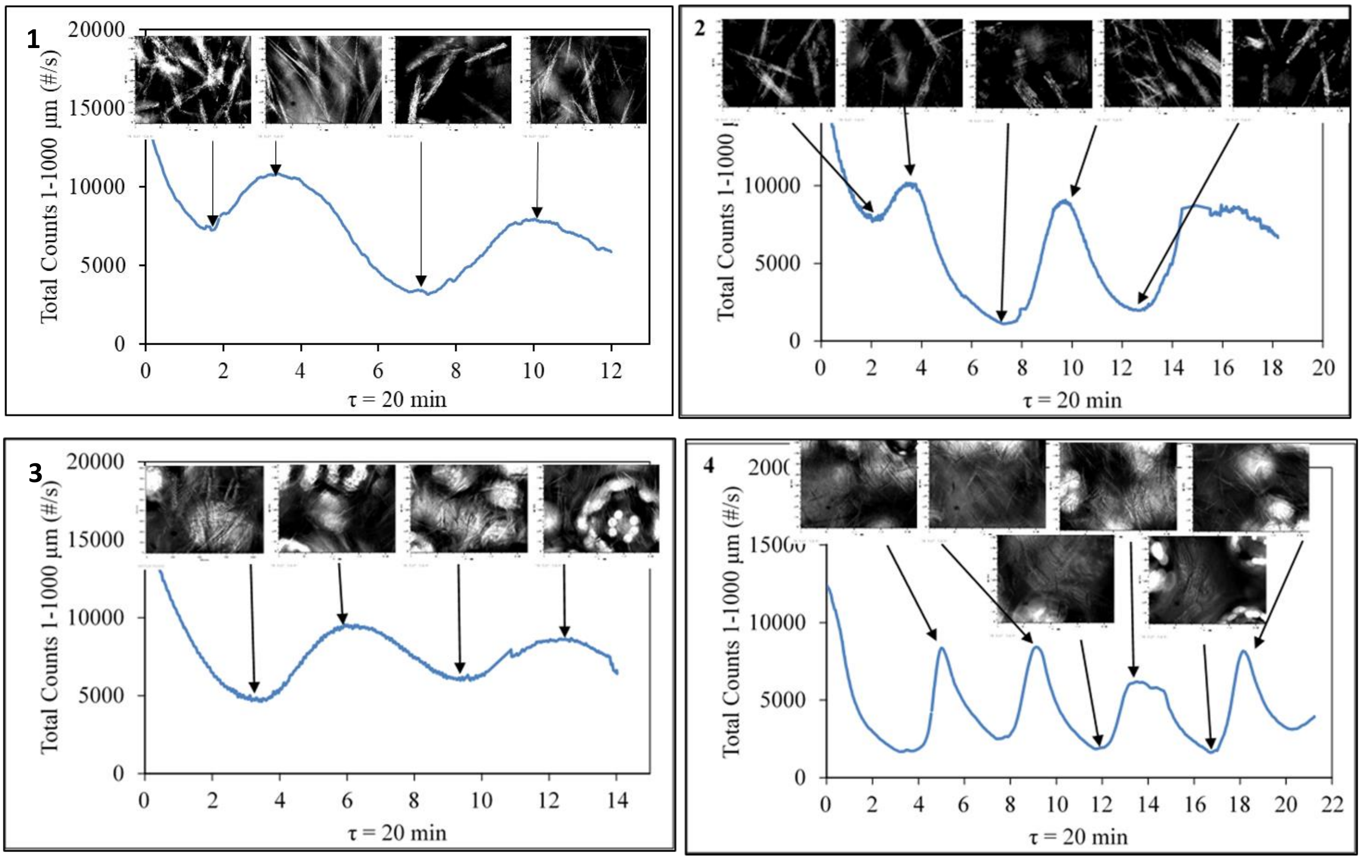

Figure S6. PVM images on the peaks and troughs throughout the MSMPR crystallization for experiments 1 to 4

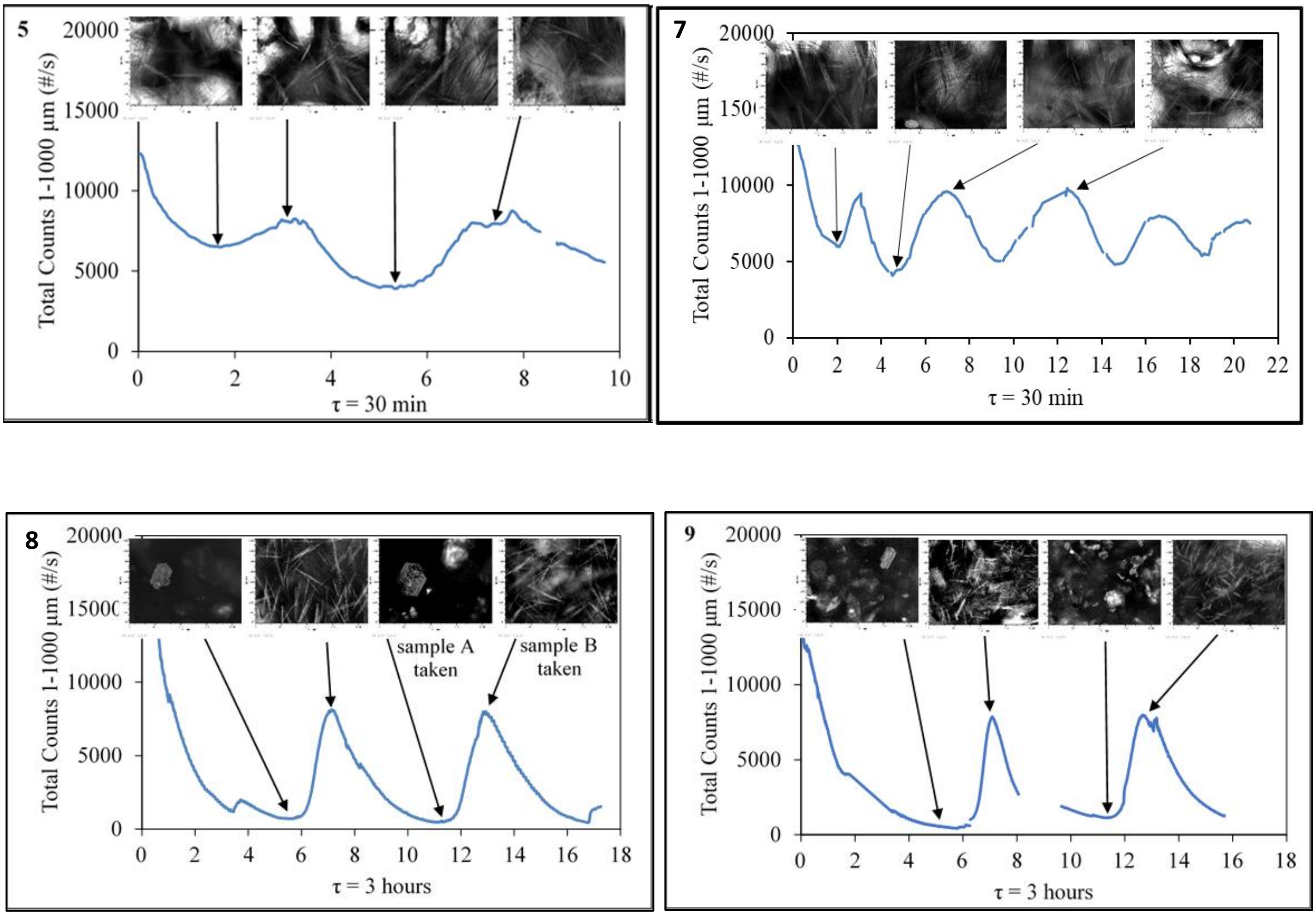

Figure S7. PVM images on the peaks and troughs throughout the MSMPR crystallization for experiments 5 - 9

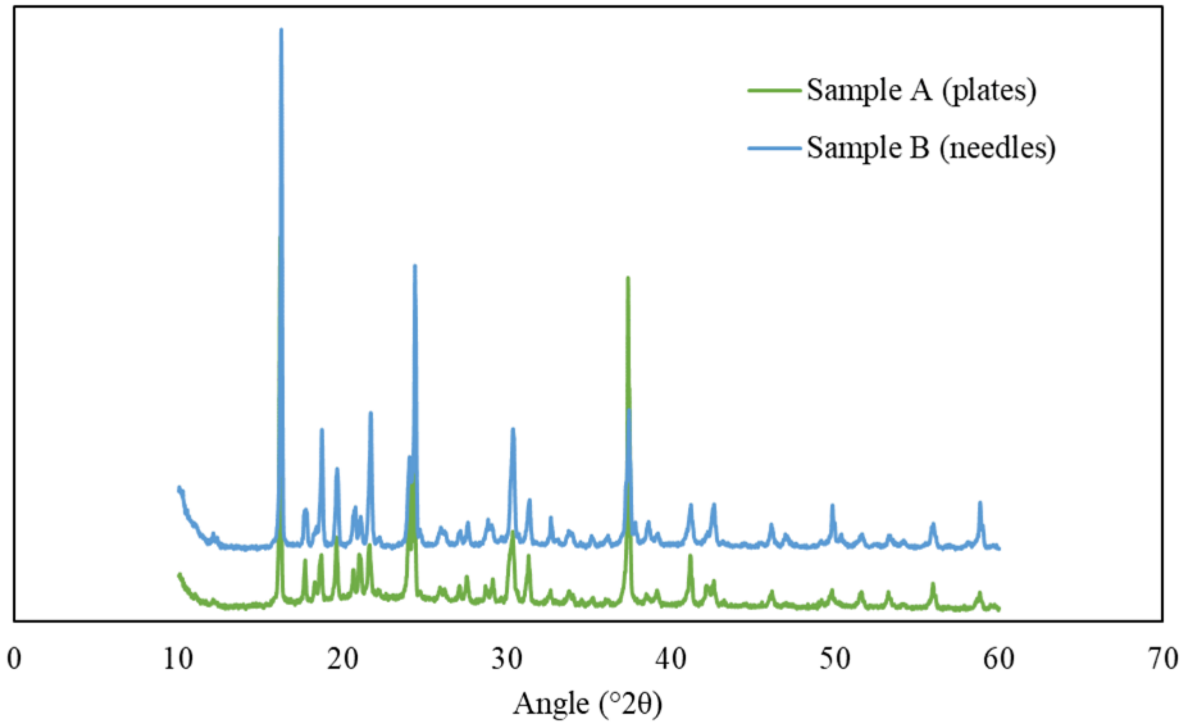

Figure S8. XRD analysis for Sample A obtained at the trough and Sample B obtained at the peak, from experiment 7, τ = 3 h at 600 rpm.

References:

1. Jones, R. C.; Twamley, B., Structure of 2-chloro-N-(p-tol-yl)propanamide. *Acta Crystallogr., Sect. E: Crystallogr. Commun.* **2018,** *74* (Pt 11), 1584-1588.
2. Pascual, G. K.; Donnellan, P.; Glennon, B.; Kamaraju, V. K.; Jones, R. C., Experimental and Modeling Studies on the Solubility of 2-Chloro-N-(4-methylphenyl)propanamide (S1) in Binary Ethyl Acetate + Hexane, Toluene + Hexane, Acetone + Hexane, and Butanone + Hexane Solvent Mixtures Using Polythermal Method. *J. Chem. Eng. Data* **2017,** *62* (10), 3193-3205.
